# National laboratory-based surveillance system for antimicrobial resistance: a successful tool to support the control of antimicrobial resistance in the Netherlands

**DOI:** 10.2807/1560-7917.ES.2017.22.46.17-00062

**Published:** 2017-11-16

**Authors:** Wieke Altorf-van der Kuil, Annelot F Schoffelen, Sabine C de Greeff, Steven FT Thijsen, H Jeroen Alblas, Daan W Notermans, Anne LM Vlek, Marianne AB van der Sande, Tjalling Leenstra, the National AMR Surveillance Study Group

**Affiliations:** 1 Centre for Infectious Diseases, Epidemiology and Surveillance, National Institute for Public Health and the Environment (RIVM), Bilthoven, The Netherlands; 2 Department of Medical Microbiology and Immunology, Diakonessenhuis, Utrecht, The Netherlands; 3 Centre for Infectious Diseases Research, Diagnostics and Screening, National Institute for Public Health and the Environment (RIVM), Bilthoven, The Netherlands; 4 The members of the National AMR Surveillance Study Group are listed at the end of the article

**Keywords:** antimicrobial resistance, surveillance, Netherlands, ISIS-AR

## Abstract

An important cornerstone in the control of antimicrobial resistance (AMR) is a well-designed quantitative system for the surveillance of spread and temporal trends in AMR. Since 2008, the Dutch national AMR surveillance system, based on routine data from medical microbiological laboratories (MMLs), has developed into a successful tool to support the control of AMR in the Netherlands. It provides background information for policy making in public health and healthcare services, supports development of empirical antibiotic therapy guidelines and facilitates in-depth research. In addition, participation of the MMLs in the national AMR surveillance network has contributed to sharing of knowledge and quality improvement. A future improvement will be the implementation of a new semantic standard together with standardised data transfer, which will reduce errors in data handling and enable a more real-time surveillance. Furthermore, the scientific impact and the possibility of detecting outbreaks may be amplified by merging the AMR surveillance database with databases from selected pathogen-based surveillance programmes containing patient data and genotypic typing data.

## Introduction

The number of infections caused by bacteria that are resistant to commonly used antimicrobials is increasing worldwide [1]. On World Health Day 2011, the Director-General of the World Health Organization (WHO) warned that the world was headed towards a post-antibiotic era [2], which she later called “*the end of modern medicine as we know it*” [3]. In 2013, the Chief Medical Officer of the United Kingdom (UK) even used the words “*apocalyptic threat*” [4].

To address this threat, an independent review committee was set up in the UK in 2014 to analyse the global problem and propose concrete actions for an international approach [5], and in 2015, the WHO prepared a global action plan [6]. In both reports, surveillance of antimicrobial resistance (AMR) was mentioned as a cornerstone in the control of AMR, but it was also stated that in many countries AMR surveillance was not yet in place or under-resourced. Therefore, several projects were set up in the past few years to increase resources for national and international AMR surveillance. Examples of these are the Central Asian and Eastern European Surveillance of AMR (CAESAR) [7] and the Global AMR Surveillance System (GLASS) [8], both developed by the WHO.

Although a good national AMR surveillance is absent in many countries, in others a well-functioning national AMR surveillance system is in place [1]. In the Netherlands, a well-functioning national AMR surveillance system has been in place since 2008. The goal of this paper is to give a comprehensive overview of this system. We describe the methodology, the output and the utilisation of the data, including methodological issues and future developments of the system. We believe that the knowledge gained from this and other systems will be useful for countries who are setting up AMR surveillance or improving an existing AMR surveillance system.

## The Dutch national antimicrobial resistance surveillance network

The Dutch national AMR surveillance system, called the ‘Infectious Diseases Surveillance Information System for Antimicrobial Resistance’ (ISIS-AR), was set up in 2008 [9-11] and designed in close collaboration with the Dutch Society for Medical Microbiology (NVMM). It is based on data from routine antimicrobial susceptibility testing (AST) in the Dutch medical microbiology laboratories (MMLs) and is maintained by the Centre for Infectious Disease Control (CIb) of the National Institute for Public Health and the Environment (RIVM). The main objectives are to monitor the magnitude and trends of AMR as well as outbreaks involving AMR. The data can be used to retrospectively identify or prospectively monitor the emergence of new AMR mechanisms based on their phenotypic expression. By providing data to support policy making in public health and healthcare, in particular the development of empiric antibiotic treatment guidelines, and to facilitate scientific research, the system contributes to quality, safety and reduction of healthcare costs.

**Figure 1:**
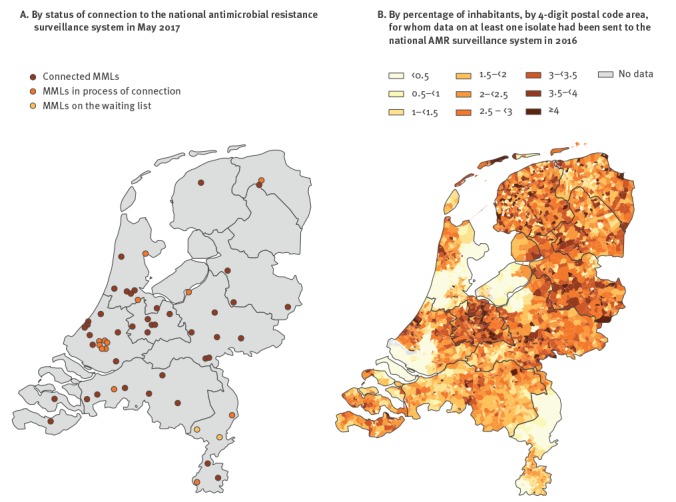
Geographical distribution of medical microbiology laboratories, the Netherlands, May 2017 (n = 57)

Participation of MMLs in the national AMR surveillance system is on a voluntary basis. Starting with eight participating MMLs in 2008, the system had grown by May 2017 into a network of 42 of 57 MMLs, distributed across the country (Figure 1A). Four of these MMLs exclusively serve a university hospital, two exclusively serve general practitioner (GP) practices, obstetrician practices, long-term care facilities and public health facilities, and 36 serve both general hospitals and GP practices. In addition, 13 MMLs are in the process of connecting to the national AMR surveillance system and the remaining two are due in the near future (Figure 1A). Figure 1B shows the regions covered by the participating MMLs in 2016 as the percentage of inhabitants, by 4-digit postal code area, for whom data on at least one isolate had been sent to the national AMR surveillance system in 2016.

MMLs: medical microbiology laboratories.

## Sampling habits and microbiological testing in the Netherlands

Because the Dutch national AMR surveillance system is entirely based on routine AST, it heavily depends on sampling policies and microbiological testing methods used in the Dutch healthcare system. Because in the Netherlands, most healthcare costs, including microbiological cultures, are covered by a mandatory health insurance scheme, sampling habits were until recently not under financial constraints. However, recent policies aimed at reducing healthcare costs that require patients to pay the first EUR 385 themselves, may cause patients to refuse diagnostics being done. To which extent these policies lead to bias in the surveillance data remains to be studied.

In hospitals, specimens for microbiological cultures are sampled for several indications: (i) diagnostic samples are usually collected from all patients presenting with signs of clinical infection, (ii) active surveillance samples are routinely collected from patients who have a high risk of developing infections with highly resistant microorganisms (HRMOs), and from patients undergoing selective gut or oropharyngeal decontamination (SDD/SOD), and (iii) screening samples are collected upon admission from patients who are at high risk of carrying an HRMO (e.g. patients transferred from a hospital in a foreign country or patients working in animal husbandry) and, if necessary, patients, family and/or personnel who had contact with a person known to be carrying an HRMO [12]. At GP practices and in nursing homes, sampling policy is more restrictive: microbiological samples are usually only collected after one or more empiric treatment failures or recurrent infection.

The majority of AST for most Gram-negative bacteria and for three Gram-positive bacteria *Staphylococcus* spp., *Enterococcus* spp., and sometimes *Streptococcus* spp. are performed using automated systems such as VITEK2 (BioMérieux, Marcy l'Etoile, France), Phoenix (Becton, Dickinson and Company, Franklin Lakes, New Jersey, United States (US)) or Microscan (Beckman Coulter, Brea, California, US). AST for other Gram-positive pathogens is mostly performed using disk diffusion or gradient tests. Between 2011 and 2013, most Dutch MMLs adapted their AST standards to match the guidelines developed by the European Committee on AST (EUCAST [13]). Previously, laboratories used various standards, predominantly those from the Clinical and Laboratory Standards Institute (CLSI [14]). HRMOs are confirmed according to laboratory detection standards of the NVMM [15]. All laboratories are involved in at least one national or international quality scheme for susceptibility testing.

## Collection of data

**Figure 2:**
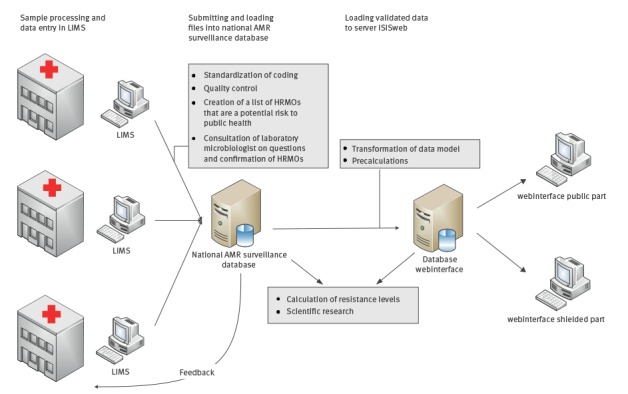
Data flow of the Dutch national antimicrobial resistance surveillance system

AMR: antimicrobial resistance; HRMO: highly resistant microorganisms; ISISweb: Infectious Diseases Surveillance Information System web interface; LIMS: laboratory information management system.

Figure 2 shows the data flow of the national AMR surveillance system. Participating MMLs are asked to provide data on all positive cultures for which antibiotic susceptibility has been determined.

Each month, AST test results of the previous month are extracted from the laboratory information management system (LIMS) in a fixed data format. The dataset contains anonymised data on all successive isolates per patient, with a patient identifier, codes for the specimen, the species identified and, for each tested agent, the susceptible/intermediate/resistant (S/I/R) interpretations as well as the crude minimum inhibitory concentrations (MIC) and zone diameters, if available. For each isolate, epidemiological data about the patient are collected, including year and month of birth, sex, healthcare setting (GP, nursing home, outpatient clinic, inpatient clinic), the indication for sampling (diagnostic, active surveillance, screening) and, for hospitalised patients, the date of admission, the hospital ward (e.g. intensive care unit) and the medical specialty of the physician requesting the diagnostics.

The multidisciplinary team at the RIVM that handles incoming data consists of data managers, epidemiologists and medical microbiologists. Before a file of incoming data is included in the surveillance database, the data have to be translated to standardised codes because different laboratories use different codes. The development and maintenance of the necessary MML-specific translation tables requires intensive alignment between surveillance team and MML, especially before the initial connection of an MML to the system. After translation of the codes, the data are subjected to a thorough quality control to identify technical deficiencies such as unmapped codes, deviations from usual patterns (e.g. in numbers of isolates or distribution of organisms), missing S/I/R interpretations or disk diameters entered in a field defining MIC values. In addition, the data are checked for unexpected results such as exceptional phenotypes as defined by EUCAST [16] and resistance proportions deviating from those in historical data from the MML. Finally, a list of HRMOs is compiled.

All findings resulting from the quality checks are, preferably within two weeks, fed back to the MML for verification by the medical microbiologist and corrected if necessary. For HRMOs, information on confirmatory tests is requested if not available in the data provided. Only after verification by the medical microbiologist are the data finalised and used for analysis. Protection of personal information is ensured by collecting and storing only anonymised data and by thorough security management of the database in compliance with the baseline information security of the Dutch government.

To illustrate the data in the database of the Dutch national AMR surveillance system, characteristics of patients and isolates from 2016 are shown by specimen type in Table 1. Most isolates (59% of 437,135 isolates) were cultured from urine. Of those, 61% were collected in GP practices, mainly from women (67%), and *Escherichia coli* was most often isolated (53%). Blood isolates were predominantly cultured from hospitalised patients, and the most frequently isolated microorganisms were *E. coli* (20%) and coagulase-negative *Staphylococcus* (40%). From the lower respiratory tract, mainly *Haemophilus influenzae* (26%) was cultured, whereas *Staphylococcus aureus* (35%) was the most common microorganism in wound and pus samples. Overall, most isolates (91%) originated from adults, especially those older than 65 years (54%).

**Table 1:** Characteristics of isolates from the Dutch national antimicrobial resistance surveillance database, 2016 (n = 437,135)

	Blood		Urine		Lower respiratory tract		Wound pus ^a^		Other sterile materials	
	n	%	n	%	n	%	n	%	n	%
Number of isolates	25,074	100	257,515	100	35,556	100	61,874	100	57,116	100
Mean number of isolates per laboratory	643	NA	6,438	NA	889	NA	1,547	NA	1,428	NA
**Pathogen**										
*Escherichia coli*	5,025	20	136,624	53	2,581	7	6,558	11	7,184	13
*Klebsiella pneumoniae*	925	4	19,520	8	1,277	4	1,411	2	1,346	2
*Enterobacter cloacae*	336	1	4,619	2	1,047	3	2,247	4	991	2
*Proteus mirabilis*	275	1	15,000	6	585	2	2,352	4	1,258	2
Other Enterobacteriaceae ^b^	1128	4	21,126	8	3,665	10	5,614	9	5,088	9
*Pseudomonas aeruginosa*	437	2	7,861	3	3,576	10	3,820	6	4,307	8
*Acinetobacter* spp.	111	0	2,680	1	399	1	1,025	2	593	1
*Haemophilus influenza*	155	1	23	0	9,276	26	486	1	1,238	2
Other non-fermenting bacteria ^c^	68	0	1,687	1	1,439	4	853	1	593	1
*Enterococcus faecalis*	692	3	20,771	8	83	0	2,988	5	1,033	2
*Enterococcus faecium*	587	2	2,562	1	102	0	1,515	2	413	1
*Staphylococcus aureus*	2,294	9	5,935	2	4,724	13	21,949	35	17,057	30
Coagulase-negative *Staphylococcus*	10,133	40	6,977	3	64	0	5,338	9	2,548	4
*Streptococcus pneumoniae*	1,577	6	52	0	3,562	10	422	1	575	1
Other Gram-positive bacteria ^d^	1,318	5	12,076	5	498	1	5,141	8	12,620	22
*Moraxella catarrhalis*	13	0	2	0	2,678	8	155	0	272	0
**Sex of patient**										
Male	13,932	56	84,702	33	20,440	57	33,251	54	23,244	41
Female	11,142	44	172,805	67	15,115	43	28,619	46	33,857	59
**Age category of patient**										
0–4 years	1,138	5	8,350	3	587	2	2,228	4	5,019	9
5–18 years	352	1	13,264	5	813	2	2,316	4	5,350	9
19–64 years	7,627	30	84,616	33	12,291	35	26,751	43	31,115	54
> 65 years	15,957	64	151,285	59	21,865	61	30,579	49	15,632	27
**Type of care**										
General practitioner	50	0	156,956	61	2,503	7	8,326	13	18,913	33
Outpatient departments	0	0	50,739	20	11,897	33	24,065	39	19,984	35
Inpatient departments (excl. intensive care units)	22,215	89	48,061	19	16,074	45	26,919	44	14,614	26
Intensive care units	2,809	11	1,759	1	5,082	14	2,564	4	3,605	6

NA: not applicable.

Only the first diagnostic isolate per patient was included.

^a^ Including sterile tissues.

^b^ *Morganella* spp., *Citrobacter* spp., *Serratia* spp., *Providencia* spp., *Enterobacter* spp., *Proteus* spp. (non-*mirabilis*), *Klebsiella* spp. (non-*pneumoniae*).

^c^ *Pseudomonas* spp. (non-*aeruginosa*), *Stenotrophomonas* spp.

^d^ *Streptococcus* spp. (non-*pneumoniae*).

## Calculation of resistance levels

**Figure 3:**
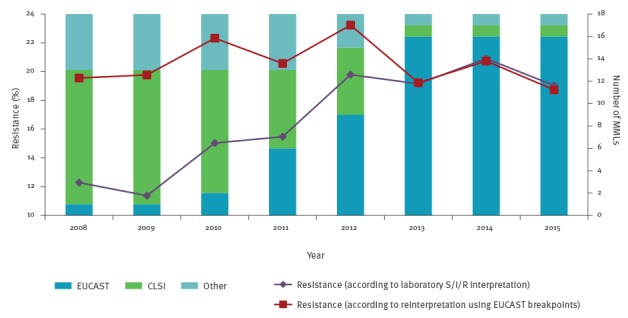
Time trends for norfloxacin resistance in *Escherichia coli*, according to laboratory S/I/R interpretation versus re-interpretation of raw testing values according to EUCAST (n = 21,382 samples) and usage of antimicrobial susceptibility testing standards (n = 18 laboratories), the Netherlands, 2008–2015

CLSI: Clinical and Laboratory Standards Institute; EUCAST: European Committee on Antimicrobial Susceptibility Testing; MMLs: medical microbiology laboratories; S/I/R: susceptible/intermediate/resistant.

Resistance levels are calculated as the proportion of all tested isolates of the same species that is resistant (R) or non-susceptible (I + R) to the antimicrobial of interest. Before resistance trends are calculated, AST results are standardised. The importance of standardisation became clear when almost all laboratories adapted their testing standards to EUCAST guidelines [13] between 2011 and 2013. Although this did not have much impact on resistance levels and trends for most bug–drug combinations [17], a distortion of trends caused by large differences in breakpoints before and after the change of guideline was seen for some combinations. This is illustrated in Figure 3, which shows a gradual increase in norfloxacin resistance in *E. coli* that is entirely due to a gradual change in the AST breakpoint standards used.

The analysis includes 18 MMLs for which continuous data over the period from 2008 to 2015 was available. S and R susceptibility breakpoints are, respectively, ≤ 4 and ≥ 16 according to CLSI [14] and ≤ 0.5 and > 1 according to EUCAST [13].

To avoid the presentation of such false time trends, crude test values, if available, have since 2012 been reinterpreted according to the latest EUCAST clinical breakpoints. If re-interpretation is not possible, the isolate is excluded from the analysis. To date, re-interpretation has been based only on MIC values from automated systems, microdilution or gradient tests because interpretation of zone diameters is heavily dependent on the disk diffusion method used, owing to differences in medium, inoculation density and amount of antibiotic in the disk. Therefore, for microorganisms mainly tested by disk diffusion, or if otherwise less than 80% of results can be re-interpreted, only cross-sectional resistance levels are calculated, based on the S/I/R interpretation from MMLs that used EUCAST AST guidelines during the whole analysed period.

To avoid overestimation of resistance caused by multiple counting [18,19], one isolate per patient is included. Depending on the objectives of the analysis, this may be the first, the last or the most resistant isolate per patient, per species, per calendar year or per estimated episode of infection [20]. If necessary, this selection can be further specified by specimen, healthcare setting and hospital ward. However, because we only have unique patient identifiers per institution, patients transferring between hospitals are counted multiple times. The extent of multiple counting in our database is unclear. To solve this issue, a countrywide unique patient identifier is necessary, for example an encrypted, and therefore anonymised, citizen service number. However, this involves privacy and data security issues that have not been solved yet.

To avoid overestimation of resistance caused by active screening for resistance, only data on diagnostic cultures are included for most research questions [19,20]. To minimise overestimation of resistance caused by second level susceptibility testing or selective testing [19,20], analyses on antimicrobial susceptibility are conducted on data from only those MMLs that tested susceptibility to the antimicrobial agent of interest in at least 50% of isolates of the species in question.

A remaining challenge is how to adjust for the influence of changes in sampling practices on the calculated level of resistance. Because resistance levels are calculated as the proportion of resistant among the total number of tested isolates, selective sampling, in which patients with resistant microorganisms are more likely to be sampled, increases the observed resistance proportions [21]. In Dutch hospitals, specimens for microbiological cultures are usually sampled whenever there is a clinical suspicion of an infection, especially suspected blood stream infection. However, because of the restrictive sampling policy in Dutch GP practices and nursing homes, the percentage of resistant isolates in these settings is expected to be overestimated. If not interpreted with caution, this may influence decisions on empiric therapy towards an unnecessarily increased use of second-line antibiotics.

## Output and utilisation of the data

Results from the national AMR surveillance system are published in several ways. Firstly, resistance proportions and temporal trends are described for a selection of clinically relevant bacterial pathogens in the annual report on consumption of antimicrobials and antimicrobial resistance in the Netherlands [22]. Secondly, resistance figures are provided by the RIVM on request or can be obtained through interactive reports on the web interface (www.ISISweb.nl). This interactive website facilitates generation of AMR reports for different specimen types, patient groups, and hospital wards. On the publicly available part of the website, only nationally aggregated data can be generated, whereas the password-protected part allows representatives of participating MMLs to generate reports for their own institution. The reports are benchmarked with nationally aggregated data from all hospitals and from hospitals of the same type as the local hospital, which is possible because of the uniformly standardised codes in the surveillance system. In addition, the protected part of the website generates standard reports every 3 months for each individual hospital, again benchmarked against national figures. Finally, a subset of the surveillance data is sent annually to the European Antimicrobial Resistance Surveillance Network (EARS-Net) which is coordinated by the European Centre for Disease Prevention and Control [23].

The information provided by the national AMR surveillance system is used in medical practice and public health in several ways. Firstly, resistance figures are used as a rational base for empiric therapy, for development of national and local antibiotic treatment guidelines and for antibiotic stewardship programmes. One example is the development of the empiric treatment guideline for general practitioners on otitis media in children [24], which used national resistance data provided following a request from the College of General Practitioners who developed that guideline. At the national level, the surveillance data help create awareness of antibiotic resistance and advocate prudent antimicrobial use, for example through the website www.volksgezondheidenzorg.info/onderwerp/antimicrobiële-resistentie-amr. Furthermore, data from the surveillance system help interpret the potential impact of new resistance mechanisms. For example, with regard to the discovery of the *mcr-1* resistance mechanism for colistin in Enterobacteriaceae [25], surveillance data showed that phenotypic resistance levels for colistin in *E. coli* and *Klebsiella pneumoniae* had remained stable during the past 5 years (Table 2). Although it is not possible to analyse trends in the distribution of the gene because only phenotypic data are collected, data from the national AMR surveillance system can be used to select relevant isolates for additional testing.

**Table 2:** Resistance levels for colistin in *Escherichia coli* and *Klebsiella pneumoniae* isolates from the national antimicrobial resistance surveillance database, the Netherlands, 2011–2015 (n = 132,116)

Year	Number of tested isolates	Colistin-resistant isolates		
		n	%	95% CI
*Escherichia coli*				
2011	19,651	71	0.4	0.3–0.4
2012	25,724	119	0.5	0.4–0.5
2013	24,882	104	0.4	0.3–0.5
2014	20,999	89	0.4	0.3–0.5
2015	20,589	105	0.5	0.4–0.6
*Klebsiella pneumoniae*				
2011	3,727	45	1.2	0.9–1.6
2012	4,485	43	1.0	0.7–1.2
2013	4,267	61	1.4	1.1–1.8
2014	3,813	39	1.0	0.7–1.3
2015	3,979	45	1.1	0.8–1.5

CI: confidence interval.

The national AMR surveillance system provides opportunities for in-depth scientific research that generates additional knowledge on AMR. Examples of research based on Dutch national surveillance data are studies on the impact the EUCAST breakpoint implementation in Dutch MMLs had on resistance levels in surveillance data [17,26], a review on the adequacy of the urinary tract infection treatment guideline in hospitalised patients [27], a trend analysis for AMR in hospitals where SDD/SOD is applied vs hospitals where this is not the case [28,29], trends in the proportion of *E. coli* and *K. pneumoniae* with an ESBL-producing gene [30], and detection and epidemiology of carbapenemase-producing Enterobacteriaceae in the Netherlands [31].

As an additional effect of national surveillance, participation of the MMLs in the national AMR surveillance network has contributed to sharing of knowledge and quality improvement. Triggered by the feedback on the monthly file, many MMLs perform more confirmatory tests for potential HRMOs and exceptional phenotypes such as carbapenem resistance in Enterobacteriaceae or vancomycin non-susceptibility in methicillin-resistant *S. aureus* (MRSA). Furthermore, quality control by the national surveillance team has revealed methodological issues with regard to AST. For example, in data from laboratories using the VITEK2 test panel P608, it was not possible to differentiate between rifampicin-susceptible and -intermediate *S. aureus* isolates according to EUCAST breakpoints because the lowest concentration in the test panel was at the R breakpoint of ≤ 0.5. In the new test panel P633, the concentration range is adapted.

Finally, the national surveillance network provides a platform for further harmonisation of AST methods in participating MMLs, which benefits the comparability of local data with nationwide data. One of the initiatives that were supported by the national surveillance network was the development of a guideline on a uniform test panel for disk diffusion in microorganisms that cannot be tested with automated systems. This guideline is intended to harmonise the test panels between laboratories and consequently provide information on resistance for both surveillance and clinical purposes.

## Future developments and challenges

To enhance quality and timeliness of data delivery to the national AMR surveillance system, the NVMM, RIVM and other stakeholders are working together to develop a new electronic messaging standard. This comprises a common data model, a new semantic standard [32] that is a subset of the existing international coding schemes SNOMED (www.ihtsdo.org/snomed-ct) and LOINC (www.loinc.org) as well as different HL7-based (www.hl7.org) data transfer messages. A number of MMLs, together with the RIVM, different LIMS vendors and other stakeholders, have started a pilot project that uses the new standards to generate and send data extractions to the database of the national surveillance system.

Data collection in the national AMR surveillance system is currently limited to phenotypic susceptibility tests and some epidemiological information. To amplify the impact of the surveillance system on public health, healthcare and scientific research, opportunities are being explored to merge data from the national AMR surveillance system with existing data on type of infection, antibiotic use, co-morbidity and mortality at patient level and/or genotypic data. This will require standardised, encrypted patient and sample identifiers in each system, but once established, it will allow insight into the risk factors, spread and burden of AMR.

Another envisioned development is the design of an algorithm for the detection of (multi-institutional) outbreaks of HRMOs. In the Netherlands, hospitals usually have a well-functioning infection prevention policy in which detection of local outbreaks is incorporated. Nevertheless, individual laboratories do not have access to data from neighbouring laboratories and analysis of data from the national AMR surveillance system can serve as a safety net to detect local or multi-institutional outbreaks. We used SaTScan software (www.SaTScan.org) to develop an algorithm to detect clusters of resistant microorganisms in space and time, but many of these clusters were found not to be clonal upon validation (data not shown). Currently, a new approach is being developed to detect clusters of resistant microorganisms, possibly in combination with other datasets. 

A remaining challenge is to develop a method to adequately distinguish between healthcare-associated and community-acquired infections. Currently, it is not possible to identify which patients are transferred from one hospital to another because the national AMR surveillance system does not use a countrywide unique patient identifier. This may lead to healthcare-associated infections being misclassified as community-acquired. Because the distinction between these types of infection is increasingly important in the context of antibiotic stewardship, it is desirable to have insight into healthcare transfers by using countrywide unique patient identifiers, which is currently not available for our system.

## Conclusion

Since 2008, the Dutch national AMR surveillance system has developed into a successful tool for the surveillance and control of AMR in the Netherlands. This was possible because of the successful collaboration between the surveillance team and the medical microbiologists from the MMLs, and because of investment in epidemiological knowledge.

The system provides background information for policy making in public health and healthcare, it supports the development of empiric antibiotic therapy guidelines and facilitates in-depth research. In addition, participation of the MMLs in the national AMR surveillance network has contributed to sharing of knowledge and quality improvement.

A future improvement to the system will be the implementation of a new semantic standard with standardised data transfer, which will reduce errors in data handling and enable more real-time surveillance. Furthermore, the impact of the system may be amplified by a possibility to merge with databases containing clinical patient data and genotypic data on microorganisms, and by development of a validated cluster detection system for institutional and multi-institutional outbreaks.

## Notes

**Members of National AMR Surveillance Study Group:** J.W.T. Cohen-Stuart, Department of Microbiology, Noordwest Ziekenhuisgroep, Alkmaar. A.J.L. Weersink, Department of Medical Microbiology, Meander Medical Centre, Amersfoort. C.E. Visser, Department of Medical Microbiology, Academic Medical Center, Amsterdam. C.M.J.E. Vandenbroucke-Grauls, Department of Medical Microbiology and Infection Control, VU University medical center Amsterdam, Amsterdam. M.L. van Ogtrop, Department of Medical Microbiology, Onze Lieve Vrouwe Gasthuis, Amsterdam. D.J. Soeltan-Kaersenhout, Department of Medical Microbiology, Slotervaart Hospital / Netherlands Cancer Institute, Amsterdam. M. Scholing, Public Health Laboratory, Public Health Service Amsterdam, Amsterdam. B.C. van Hees, Department of Medical Microbiology and Infection prevention, Gelre Hospitals, Apeldoorn. P.H.J. van Keulen, Department of Medical Microbiology, Bravis Hospital, Bergen op Zoom. J. Alblas, W. Altorf-van der Kuil, L. Blijboom, F.N.J. Frakking, S.C. de Greeff, S. Groenendijk, J. van Heereveld, R. Hertroys, J.C. Monen, D.W. Notermans, A.F. Schoffelen, M.I. van Triest, A.L.M. Vlek, C.C.H. Wielders, S.H.S. Woudt, Centre for Infectious Diseases, Epidemiology and Surveillance. National Institute for Public Health and the Environment (RIVM), Bilthoven. P.H.J. van Keulen, J.A.J.W. Kluytmans, Laboratory for Microbiology and Infection Control, Amphia Hospital, Breda. E.M. Kraan, Department of Medical Microbiology, IJsselland hospital, Capelle a/d Ijssel. E.E. Lundblad-Mattsson, Department of Medical Microbiology, Reinier de Graaf Groep, Delft. F.W. Sebens, Department of Medical Microbiology, Deventer Hospital, Deventer. E. de Jong, Department of Medical Microbiology, Slingeland Hospital, Doetinchem. H.M.E. Frénay, B. Maraha, Department of Medical Microbiology, Albert Schweitzer Hospital, Dordrecht. A.J. van Griethuysen, Department of Medical Microbiology, Gelderse Vallei Hospital, Ede. W. Silvis, Laboratory of Medical Microbiology and Public Health, Enschede A. Demeulemeester, SHL-Groep, Etten-Leur. B.B. Wintermans, Department of Medical Microbiology, Admiraal De Ruyter Hospital, Goes. M.J.C.A. van Trijp, Groene Hart Ziekenhuis Gouda, Gouda. M.G.R. Hendrix, A. Ott, Department of Medical Microbiology, Certe, Groningen. J.P. Arends, G.A. Kampinga, Department of Medical Microbiology, University of Groningen, Groningen. D. Veenendaal, Regional Laboratory of Public Health, Haarlem. A.J.L. Weersink, Department of Medical Microbiology, St Jansdal Hospital, Harderwijk. E.I.G.B. de Brauwer, F.S. Stals, Department of Medical Microbiology and Infection Control, Zuyderland Medical Centre Heerlen, Heerlen. L.J. Bakker, J.W. Dorigo-Zetsma, Department of Medical Microbiology, CBSL, Tergooi Hospital, Hilversum. B. Ridwan, Department of Medical Microbiology, Westfriesgasthuis, Hoorn. J.H. van Zeijl, Izore, Centre for Infectious Diseases Friesland, Leeuwarden. A.T. Bernards, Department of Medical Microbiology, Leiden University Medical Center, Leiden. S. Erkens-Hulshof, Department of Medical Microbiology, Maastricht University Medical Centre, Maastricht. B.M. de Jongh, Department of Medical Microbiology and Immunology, St Antonius Hospital, Nieuwegein. M.H. Nabuurs-Franssen, Department of Medical Microbiology and Infectious Diseases, Canisius Wilhelmina Hospital, Nijmegen. S. Kuipers, Department of Medical Microbiology, Radboud University Nijmegen Medical Center, Nijmegen. B.M.W. Diederen, Department of Medical Microbiology, Bravis Hospital, Roosendaal. D.C. Melles, Department of Medical Microbiology, Erasmus University Medical Center, Rotterdam. M. van Rijn, Department of Medical Microbiology, Ikazia Hospital, Rotterdam.P. de Man, Department of Medical Microbiology, Sint Franciscus Gasthuis, Rotterdam. D.C. Melles, STAR Medical Diagnostic Center Rotterdam, Rotterdam. N. Vaessen, Department of Medical Microbiology, Franciscus Vlietland Hospital, Schiedam. M.A. Leverstein - van Hall, Department of Medical Microbiology and Infection Control, Alrijne Hospital, Leiden/ HMC, The Hague. E.P.M. van Elzakker, Department of Medical Microbiology, Haga Teaching Hospital, The Hague. A.E. Muller, Department of Medical Microbiology, MCH Westeinde Hospital, The Hague. N.H. Renders, Department of Medical Microbiology and Infection Control, Jeroen Bosch Hospital, 's-Hertogenbosch. D.W. van Dam, Department of Medical Microbiology and Infection Control, Zuyderland Medical Centre, Sittard-Geleen. B.G.A. Hendrickx, Department of Medical Microbiology, ZorgSaam Hospital Zeeuws-Vlaanderen, Terneuzen. A.G.M. Buiting, Department of Medical Microbiology, St. Elisabeth Hospital, Tilburg. M.P.D. Deege, Department of Medical Microbiology, Saltro Diagnostic Centre, Utrecht. F.N.J. Frakking, Department of Medical Microbiology, University Medical Centre Utrecht, Utrecht. A.L.M. Vlek, Department of Medical Microbiology and Immunology, Diakonessenhuis, Utrecht. I.T.M.A. Overdevest, Department of Medical Microbiology, PAMM, Veldhoven. R.W. Bosboom, Laboratory for Medical Microbiology and Immunology, Rijnstate Hospital, Velp. T.A.M. Trienekens, VieCuri Medical Center Venlo, Venlo. G.P. Voorn, Department of Medical Microbiology, Zuwe Hofpoort Hospital, Woerden. G.J.H.M. Ruijs, M.J.H.M. Wolfhagen, Laboratory of Medical Microbiology and Infectious Diseases, Isala, Zwolle.
